# Effect of Edge-Preserving Adaptive Image Filter on Low-Contrast Detectability in CT Systems: Application of ROC Analysis

**DOI:** 10.1155/2008/379486

**Published:** 2008-11-16

**Authors:** Miwa Okumura, Takamasa Ota, Kazuhisa Kainuma, James W. Sayre, Michael McNitt-Gray, Kazuhiro Katada

**Affiliations:** ^1^CT Systems Development Department, Toshiba Medical Systems Corporation, 1385 Shimoishigami, Otawara-Shi, Tochigi 324-8550, Japan; ^2^Embedded Systems Solutions Division, Toshiba Information Systems (Japan) Corporation, 1-53 Nissin-Cho, Kawasaki-Ku, Kawasaki-Shi, Kanagawa 210-8540, Japan; ^3^Department of Radiological Sciences, UCLA Medical center, David Geffen School of Medicine, 924 Westwood Bouelvard, Suite 650 Los Angeles, CA 90024, USA; ^4^Department of Radiology, Fujita Health University School of Medicine, 1-98 Dengakugakubo, Kutsukake-Cho, Toyoake-Shi, Aichi 470-1192, Japan

## Abstract

*Objective*. For the multislice CT (MSCT) systems with a larger number of detector rows, it is essential to employ dose-reduction techniques. As reported in previous studies, edge-preserving adaptive image filters, which selectively eliminate only the noise elements that are increased when the radiation dose is reduced without affecting the sharpness of images, have been developed. In the present study, we employed receiver operating characteristic (ROC) analysis to assess the effects of the quantum denoising system (QDS), which is an edge-preserving adaptive filter that we have developed, on low-contrast resolution, and to evaluate to what degree the radiation dose can be reduced while maintaining acceptable low-contrast resolution. *Materials and Methods*. The low-contrast phantoms (Catphan 412) were scanned at various tube current settings, and ROC analysis was then performed for the groups of images obtained with/without the use of QDS at each tube current to determine whether or not a target could be identified. The tube current settings for which the area under the ROC curve (Az value) was approximately 0.7 were determined for both groups of images with/without the use of QDS. Then, the radiation dose reduction ratio when QDS was used was calculated by converting the determined tube current to the radiation dose. *Results*. The use of the QDS edge-preserving adaptive image filter allowed the radiation dose to be reduced by up to 38%. *Conclusion*. The QDS was found to be useful for reducing the radiation dose without affecting the low-contrast resolution in MSCT studies.

## 1. INTRODUCTION

Compared with conventional X-ray CT systems, the time required for scanning various anatomical regions with various slice thicknesses has been markedly reduced due to the introduction of multislice CT (MSCT) systems with a larger number of detector rows. However, since it is now possible to scan a wide range with a thin slice thickness during a single breath-hold, there has been an increasing concern regarding the higher radiation doses in a wide range of clinical applications. In addition, due to the high-speed scanning capabilities of the latest MSCT scanners that allow the same range to be scanned repeatedly at short intervals, further efforts in the area of image processing must be made to reduce the radiation dose in order to fully exploit the benefits of MSCT. As the dose is reduced, image noise is increased. A lowpass filter processing can reduce image noise, but the edges of objects become less clear and the sharpness of images is reduced. To address these issues, the development of edge-preserving adaptive filters, which selectively eliminate only the noise elements that are increased when the radiation dose is reduced without affecting edge intensity, has been reported [1–4].

We have developed the quantum denoising system (QDS), which is one type of edge-preserving adaptive filter, and have assessed its physical characteristics [5]. QDS is an adaptive filter that extracts the three-dimensional edge intensity for each pixel and adjusts the amount of smoothing according to the edge intensity. Specifically, the amount of smoothing is increased in uniform regions with little edge content in order to reduce image noise and the amount of smoothing is reduced in regions near stronger edges so that the edges are maintained. In a previous study [5], we reported the physical characteristics of QDS, for example, relationships between edge intensity and frequency response (modulation transfer function; MTF), and noise characteristics (noise power spectrum; NPS). It was shown that the MTF curve varied smoothly according to the edge intensity and that the noise reduction characteristics obtained in QDS-processed images were comparable to those obtained in Gaussian filter-processed images.

In the present study, we evaluated the effectiveness of the QDS using receiver operating characteristic (ROC) analysis [6–12] from viewpoint of radiation dose reduction. ROC analysis was employed for the objective evaluation of QDS in order to evaluate how far the dose can be reduced with QDS while maintaining an acceptable low-contrast resolution.

## 2. MATERIALS AND METHODS

### 2.1. QDS algorithm

The quantum denoising system (QDS) is a three-dimensional edge-preserving adaptive filter that has been developed [5] which aims to reduce noise while maintaining the spatial resolution in the *XY* plane without increasing the effective slice thickness in the *Z* direction. First, smoothing processing is performed on the input image using a lowpass filter to reduce high-frequency noise elements, while sharpening processing is performed on the input image using a highpass filter to enhance fine structures. The smoothed image and the sharpened image are defined as Smooth(*x*, *y*, *z*) and Sharp(*x*, *y*, *z*), respectively. The edge elements that should be maintained are expressed by the following formula for each pixel:

(1) Edge(x, y, z) =|Sharp(x, y, z)−Smooth(x, y, z)|.

The edge elements are then converted to values between 0 and 1 based on the edge sensitivity curve and used to calculate the appropriate blending ratio for Smooth(*x*, *y*, *z*) and Sharp(*x*, *y*, *z*). The edge sensitivity curve *w*(Edge(*x*, *y*, *z*)) is expressed by the following formula using a sigmoid function:

(2) $$w\left(\text{Edge}(x,\;y,\;z)\right)=\frac{\left(\frac{\left(e^{\left(1/\alpha )\cdot (\text{Edge}(x,y,z)-\beta \right)}\;-\;e^{\left(-1/\alpha )\cdot (\text{Edge}(x,y,z)-\beta \right)}\right)}{\left(e^{\left(1/\alpha )\cdot (\text{Edge}(x,y,z)-\beta \right)}\;+\;e^{\left(-1/\alpha )\cdot (\text{Edge}(x,y,z)-\beta \right)}\right)}+1\right)}{2}.$$

**Figure 1:**
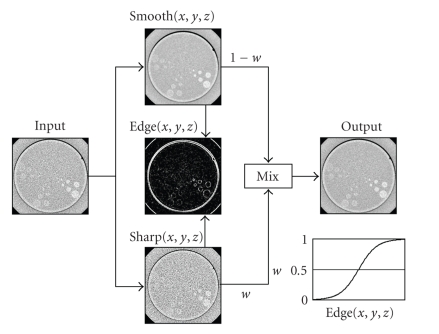
Quantum denoising system (QDS) algorithm. The results of smoothing filtering and sharpening filtering are blended together based on the edge intensity.

In this formula, *α* is a parameter indicating the gradient of the rising slope of the curve and *β* is a parameter indicating the threshold value of the target edge intensity. Both are determined based on the CT number distribution for each anatomical region. As shown in Figure 1, the blending ratio *w*(Edge(*x*, *y*, *z*)) is close to 0 when the edges in the image are soft and only noise elements are present, while it is close to 1 when the edges in the image are sharp and many fine structures are present. By defining the blending formula for Smooth(*x*, *y*, *z*) and Sharp(*x*, *y*, *z*) obtained by QDS as Smooth(*x*, *y*, *z*)·(1 − *w*(Edge(*x*, *y*, *z*)) + Sharp(*x*, *y*, *z*)·*w*(Edge(*x*, *y*, *z*)), noise elements are reduced by increasing the blending ratio of the smoothed image in areas with low edge intensity, while fine structures are maintained by increasing the blending ratio of the sharpened image in areas with high edge intensity.

### 2.2. ROC analysis

**Figure 2:**
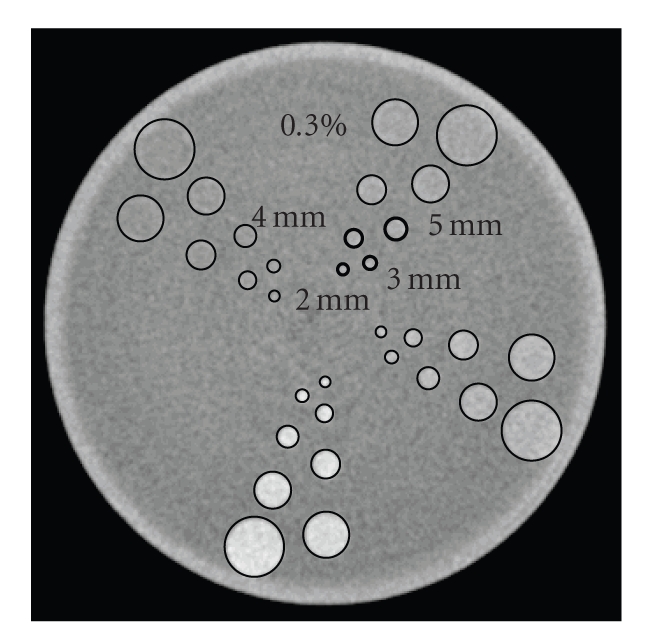
Appearance of the low-contrast phantom and locations of targets. Four types of targets (enclosed in thick lines) were used for assessment.

A 64-slice CT scanner (Aquilion; Toshiba Medical Systems Corporation, Otawara, Tochigi, Japan) was used to scan low-contrast phantoms (Catphan 412, CTP263 module; Phantom Laboratory, NY, USA). Four types (0.1%, 0.3%, 0.5%, and 1.0%) of low-contrast targets of eight sizes (2 mm, 3 mm, 4 mm, 5 mm, 7 mm, 9 mm, 12 mm, and 15 mm in diameter) are embedded in the CTP263 module. Of these 32 targets, four 0.3% low-contrast targets with diameters of 2 mm, 3 mm, 4 mm, and 5 mm were used for assessment. The appearance of the CTP263 module and the locations of the targets are shown in Figure 2. The scan conditions were 120 kV, 1 s/rot., and nonhelical scanning. The tube current was adjusted to 12 settings (320 mA, 300 mA, 250 mA, 200 mA, 180 mA, 160 mA, 140 mA, 120 mA, 110 mA, 100 mA, 90 mA, and 80 mA) and 40 images were obtained at each tube current. The scan field of view (FOV) was 240 mm. The image reconstruction conditions were as follows. The standard abdominal function (FC13) was used as the reconstruction kernel, and 8-mm-slice images were obtained by stacking four 2-mm-slice images. The reconstruction FOV was 200 mm based on the external dimensions of the Catphan phantom.

**Figure 3:**
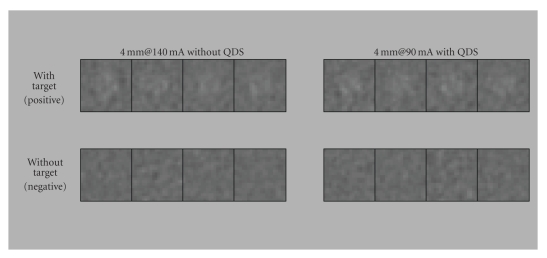
Examples of images extracted for image interpretation. The top row shows images containing targets and the bottom row shows images containing only noise elements. It should be noted that these images are examples intended to aid understanding, not images that were actually used for image interpretation.

The 40 images obtained at each tube current were then QDS-processed to generate QDS-processed images. In addition, limited regions including only one target and regions including only noise elements were extracted from each image to generate target-positive and target-negative images. The number of images for image interpretation was 80 images for each set of conditions (target size, tube current, and with/without QDS). To reduce the number of images for image interpretation, the pilot study was conducted to initially determine the appropriate three tube current settings for each target size. By performing this pilot study, the total number of images for image interpretation was reduced to 1920 images (80 images × 4 target sizes × 3 tube current settings × with/without QDS). For image interpretation, 80 images in the image group with the same conditions (same target size, same tube current, and with/without QDS) were presented in a randomized order to each observer, and image interpretation was repeated for the number of conditions that were present. Examples of images extracted for image interpretation are shown in Figure 3.

Image interpretation was performed by five radiologists with 10 to 15 years of clinical experience, and the rating of the confidence level was performed by marking the obtained confidence level on a continuous scale bar using the continuous confidence rating method [11]. It should be noted that before the main experiment, the 40 extracted images were provided to each observer in order to practice image interpretation. These images were representative images for which the Az value for all observers was expected to be approximately 0.7 and were not used for the main experiment.

The data for the rated confidence levels was input into the ROC analysis program (ROCKIT) developed by Metz at the University of Chicago, and the binormal ROC curves and parameters were estimated. The latest version of the ROCKIT program, which includes DBM MRMC analysis, can be obtained from http://www-radiology.uchicago.edu/krl/KRL_ROC/software_index.htm (accessed March 1, 2008).

ROC analysis was performed to determine the tube current settings for which the mean Az value just exceeds 0.7 from the three tube current settings selected by the pilot study for each image group with/without the use of QDS. If the mean Az value was lower than 0.6 or greater than 0.8 for a certain target size, the image interpretation experiment was performed again after shifting the tube current one level higher/lower for only that target size. The threshold value (Az = 0.7) was selected so that it would be rather difficult to determine whether or not a tumor could be identified in clinical practice [9]. MRMC analysis was used to test differences between the mean Az values for different tube currents, and the 95% confidence intervals and *P*-values were then calculated.

## 3. RESULTS

**Figure 4:**
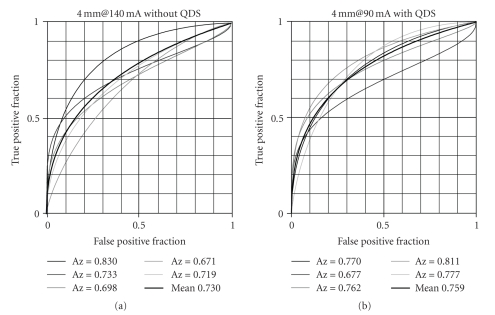
The ROC curves of five observers for images with/without the use of QDS for the target with a diameter of 4 mm. The low-contrast resolution for the images obtained at 140 mA without QDS was statistically equivalent to that of the images obtained at 90 mA with QDS.

The ROC curves of five observers for images with/without the use of QDS at different tube current settings were obtained for the 0.3% low-contrast targets of all sizes (2, 3, 4, and 5 mm). An example of the ROC curves is shown in Figure 4 for the 4 mm target. Figure 4(a) shows the ROC analysis results for images obtained at 140 mA without the use of QDS, and Figure 4(b) shows the ROC analysis results for images obtained at 90 mA with the use of QDS.

For the 4 mm target images without QDS, the mean Az value was 0.682 (<0.7) at 120 mA, 0.730 (>0.7) at 140 mA, and 0.851 (≫0.7) at 160 mA, with the mean Az value just exceeding 0.7 when the tube current was set at 140 mA. On the other hand, the mean Az value was 0.632 (<0.7) at 80 mA, 0.759 (>0.7) at 90 mA, and 0.765 (≫0.7) at 100 mA for the images obtained with the use of QDS, with the mean Az value exceeding 0.7 when the tube current was set to 90 mA. These results show that the mean Az values just exceeding 0.7 were 0.730 for images obtained at 140 mA without QDS and 0.759 for images obtained at 90 mA with QDS.

Similar results were obtained for each of the other target sizes. For the 2 mm target, the mean Az values just exceeding 0.7 were 0.709 for images obtained at 320 mA without QDS and 0.760 for images obtained at 200 mA with QDS. For the 3 mm target, the mean Az values just exceeding 0.7 were 0.706 for images obtained at 180 mA without QDS and 0.704 for images obtained at 120 mA with QDS. For the 5 mm target, the mean Az values just exceeding 0.7 were 0.717 for images obtained at 100 mA without QDS and 0.721 for images obtained at 80 mA with QDS. These results are shown in Table 1.

### 3.1. MRMC analysis

The MRMC statistical analysis [12–15] was used to test whether or not there were statistically significant differences between the images acquired with QDS and the images acquired without QDS for different tube current settings for which the mean Az value just exceeds 0.7. Specifically, the 95% confidence intervals of the differences in average Az values between the images with QDS and those without QDS were calculated and the *P*-values were then calculated for the null hypothesis that there were no differences in the mean Az values. The results are also included in Table 1. In all cases, “0” was included in the 95% confidence intervals and the *P*-values were large (>.4). It was therefore concluded that statistically significant differences could not be detected for the mean Az values between the images with QDS and those without QDS for different tube currents and the results shown in Table 1 were reasonable.

### 3.2. Dose reduction

Given that the conditions in Table 1 were found to be statistically equivalent in terms of this low-contrast performance task, the dose reduction for each condition (target size and tube current setting) from the use of QDS was calculated. Table 2 shows the tube current settings, resulting CTDI_100_ values, and dose reduction possible from using the QDS filter for the 0.3% low-contrast targets at which the statistically equivalent performance values were observed for images obtained with/without the use of QDS. These values ranged from 38% dose reduction for the 2 mm targets to 20% for the 5 mm targets.

## 4. DISCUSSION

Table 2 shows the values obtained by converting the tube current (mA) to dose (CTDI_100_, mGy). The results showed that for the target with a diameter of 2 mm, the low-contrast resolution at 27.8 mGy with the use of QDS was equivalent to that at 44.5 mGy without the use of QDS, corresponding to a dose reduction ratio of 38%. In addition, for the target with a diameter of 3 mm, the low-contrast resolution at 16.7 mGy with the use of QDS was equivalent to that at 25.0 mGy without the use of QDS, corresponding to a dose reduction ratio of 33%. Similarly, the dose reduction ratio was 36% for the target with a diameter of 4 mm and 20% for the target with a diameter of 5 mm. It should be noted that since the dose is proportional to the tube current, the dose values shown in Table 2 were obtained by performing proportional conversion based on the dose (CTDI_100_) of 44.5 mGy at 320 mA.

In the present study, the tube current settings for which the Az values exceeded 0.7 were determined from the groups of images obtained with/without the use of QDS, and it was assumed that the low-contrast resolution values at the dose for the determined tube current settings were equivalent. Then, the dose reduction ratio with the use of QDS was calculated from the tube current settings. A previous study concerning adaptive filters using ROC analysis reported that the Az values were improved when the adaptive filters were applied to images obtained at the same tube current and discussed how far the dose can be reduced [1]. We have expanded their results for the combination of various target sizes and various tube current settings, and performed statistical analysis using MRMC methods.

One limitation in this study was that we used a low-contrast resolution phantom (Catphan 412) where several uniform targets are embedded in a uniform background because the purpose of the present study was to convert the effects of the edge-preserving adaptive filters to digital form from the viewpoint of dose reduction. In the sense that system performance was to be evaluated objectively, we used a Catphan phantom with high reproducibility, which is widely employed for the evaluation of low-contrast resolution. In clinical practice, low-contrast detectability depends on the structure of the imaging target and surrounding anatomic (and pathologic) structures. Different organs may have different low-contrast detectabilities even with the same condition. Therefore, further study should investigate more complex target and background structures that simulate specific clinical cases.

The use of the QDS edge-preserving adaptive filter allowed the dose to be reduced by 38%, 33%, 36%, and 20% for 0.3% low-contrast targets with diameters of 2 mm, 3 mm, 4 mm, and 5 mm, respectively. It is therefore concluded that QDS is a significant advance in the field of image processing and that it is useful for reducing the dose in MSCT studies, in which the higher doses are an important concern.

## Figures and Tables

**Table 1:** 95% confidence intervals of the differences in average Az values and *P*-values obtained using MRMC analysis for low-contrast detection task for each target size (2, 3, 4, 5 mm) using images with and without QDS filter. These results also list the tube current used for each condition (target size, with or without QDS filter) to obtain a mean Az value that just exceeded 0.7. In all cases, “0” was included in the 95% confidence intervals and the *P*-values were large.

Target diameter	QDS	Tube current	Mean Az value	Difference	95% confidence interval		*P*-value
2 mm	Without	320 mA	0.709	−0.051	−0.176	0.074	0.410
	With	200 mA	0.760				
3 mm	Without	180 mA	0.706	0.002	−0.074	0.078	0.911
	With	120 mA	0.704				
4 mm	Without	140 mA	0.730	−0.029	−0.133	0.075	0.566
	With	90 mA	0.759				
5 mm	Without	100 mA	0.717	−0.004	−0.080	0.072	0.880
	With	80 mA	0.721				

**Table 2:** Comparison of the tube current necessary for each condition (target size, with or without QDS filter) to obtain approximately equivalent mean Az values. The last column shows the dose reduction possible with QDS for each target size at statistically equivalent performance levels based on mean Az values.

	Without QDS			With QDS			
Target diameter	Tube current	Mean Az value	Exposure dose	Tube current	Mean Az value	Exposure dose	Dose reduction ratio
2 mm	320 mA	0.709	44.5 mGy	200 mA	0.760	27.8 mGy	38%
3 mm	180 mA	0.706	25.0 mGy	120 mA	0.704	16.7 mGy	33%
4 mm	140 mA	0.730	19.5 mGy	90 mA	0.759	12.5 mGy	36%
5 mm	100 mA	0.717	13.9 mGy	80 mA	0.721	11.1 mGy	20%
